# Hyaline cartilage at the portal plate and gallbladder in biliary atresia

**DOI:** 10.4322/acr.2024.481

**Published:** 2024-04-04

**Authors:** Sangamitra Rajasekaran, Hari Neupane, Monika Bawa, Uma Nahar Saikia, Sadhna Lal, Suvradeep Mitra

**Affiliations:** 1 Post Graduate Institute of Medical Education and Research (PGIMER), Department of Histopathology, Chandigarh, India; 2 Post Graduate Institute of Medical Education and Research (PGIMER), Department of Pediatric Surgery, Chandigarh, India; 3 Post Graduate Institute of Medical Education and Research (PGIMER), Department of Pediatric Gastroenterology, Hepatology, and Nutrition, Chandigarh, India

**Keywords:** Biliary atresia, Hyaline cartilage, Portal plate, Gallbladder, Metaplasia

## Abstract

Biliary atresia (BA) is a fibro-obliterative cholestatic disease of infancy. The presence of cartilage in the resected tissue is an uncommon finding.

We documented the presence of both mature and immature hyaline cartilage in the portal plate and the wall of the gallbladder in a 2-month-old girl infant with BA who had undergone Kasai portoenterostomy.

The presence of cartilage could be part of a heterotopia or an uncommon connective tissue metaplasia. The presence of immature cartilage with the merging of the perichondrium with the soft tissue highlights a metaplastic etiology in the index case.

## INTRODUCTION

Biliary atresia (BA) is a progressive fibroinflammatory obliterative cholangiopathy of infancy that affects the biliary tree. The extrahepatic biliary tree in BA shows a surgically correctable anomaly, a procedure that reverts partially or halts the progression of liver disease. Kasai portoenterostomy (KPE) surgery is the treatment of choice in BA. Histopathologists play a main role in light microscopic examination of the extrahepatic biliary tree, including the portal plate, peribiliary glands, gallbladder, and measurement of the maximum dimension of the biliary tree. Besides the usual findings, a few uncommon discoveries are also observed in and around the extrahepatic biliary tree. Squamous, pancreatic, and cartilaginous metaplasia are uncommon findings concerning the extrahepatic biliary tree.^1,2^ A few case reports have been documented describing the presence of mature/immature cartilage in the portal tissue; however, no clinical or prognostic significance has been reported. Postulated mechanisms include metaplastic response to inflammatory insult, developmental malformation, or a simple choristoma. We document the presence of hyaline cartilage in the portal plate as well as in the wall of the gallbladder in a 2-month-old infant with BA.

## CASE REPORT

A 2-month-old infant girl presented with icterus and clay-colored stool since birth. The child also developed pruritus, upper respiratory tract infection, and diarrhea on D28 of life. She was lethargic and failed to thrive. She was a term baby, appropriate for gestational age, but was microcephalic. She did not have any other syndromic features like heterotaxy. The child had icterus and coarse crepitations in bilateral lungs on auscultation. Abdomen was distended with firm hepatosplenomegaly. Hemoglobin, total leucocyte count, and platelet counts were within normal range. Liver function tests were significantly deranged with total serum bilirubin- 12.4 mg/dL (normal range: 0.2-1.2 mg/dL), direct bilirubin- 6.3 mg/dl (normal range: 0-0.3 mg/dL), alanine aminotransferase- 191 U/L (normal range: 5-40 U/L), aspartate aminotransferase- 673 U/L (normal range: 5-40 U/L), alkaline phosphatase- 232 U/L (normal range: 42-128 U/L), total proteins-5.7 g/dL (normal range: 6.4-8.3 g/dL), and albumin- 3.74 g/dL (normal range: 3.4-4.8 g/dL). Viral markers, including CMV, were negative. Abdominal ultrasound showed an atretic gallbladder of 10mm in length with an irregular outline. There was no dilatation of the intrahepatic biliary radicals.

Kasai portoenterostomy (KPE) was performed. A wedge biopsy of liver measuring 20x12x5mm, an excised portal plate measuring 3x2x1mm, and an extra hepatic biliary tree with the atretic gallbladder measuring 15mm in length were sent for histopathological evaluation.

**Figure 1:**
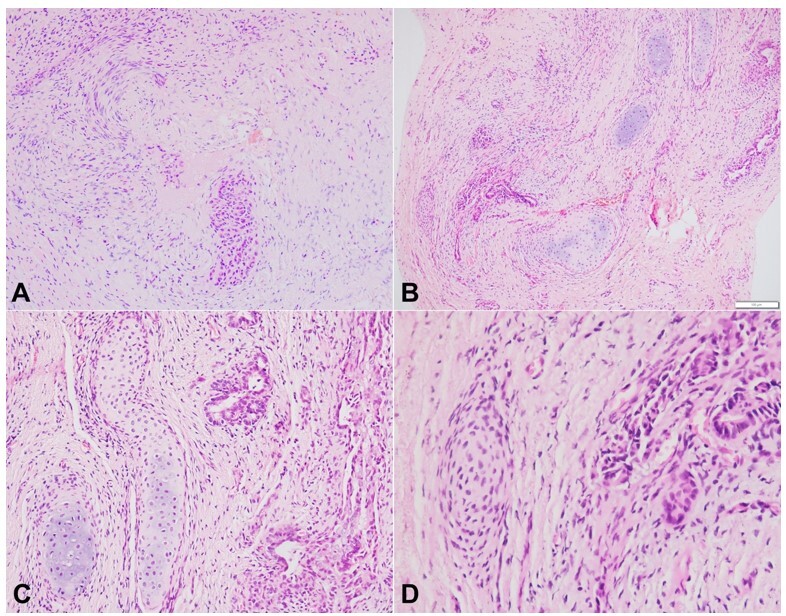
Photomicrographs of the porta hepatis: **A -** histology of extrahepatic bile duct at porta highlighting focal obliteration with periductal fibrosis, while it was focally patent (duct at the bottom of picture) (H&E, 40x); **B -** multiple islands of mature and immature hyaline cartilage in the vicinity of peribiliary glands (H&E, 40x); **C -** the mature hyaline cartilage had a basophilic matrix and thick perichondrium (H&E, 200x); **D -** the immature hyaline cartilage was hypercellular with pale stroma and poorly defined perichondrium (H&E, 400x).

The portal plate highlighted the presence of the extrahepatic bile duct with periductal edema and fibrosis. The extrahepatic bile duct measured 160.32µm in its largest dimension. The peribiliary glands around the extrahepatic bile duct showed periglandular edema and concentric fibrosis (Figure 1A). Multiple islands of mature and occasional islands of immature hyaline cartilage were noted in close approximation with the peribiliary glands (Figure 1B). The mature cartilage had well-defined perichondrium, relatively low cellularity, and basophilic chondroid matrix (Figure 1C). In contrast the immature cartilage had poorly circumscribed perichondrium merging into the adjacent soft tissue, hypercellularity, and pale matrix (Figure 1D).

**Figure 2:**
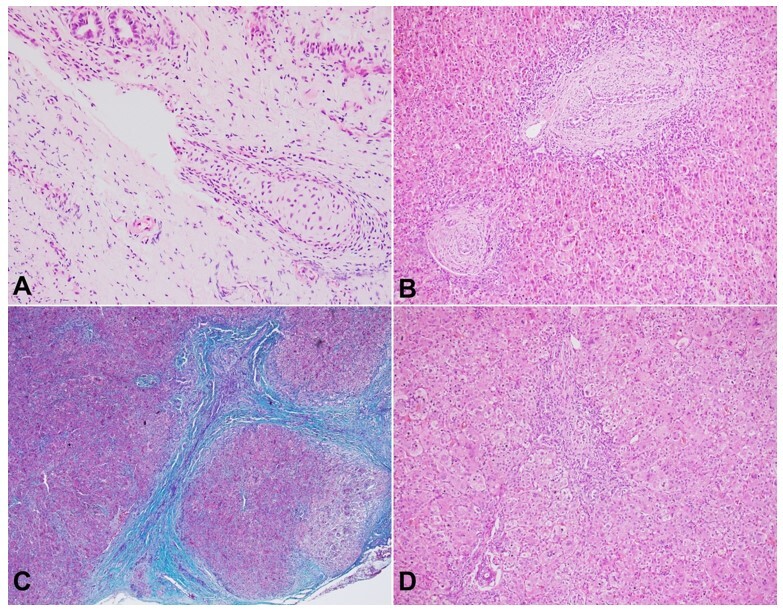
Photomicrographs of the gallbladder and the liver: **A -** the mature hyaline cartilage at the gallbladder wall with thick perichondrium (H&E, 200x); **B -** liver wedge highlighting fibro edematous and expanded portal tracts with exuberant ductular reaction (H&E, 100x); **C -** and occasional porto-portal bridging fibrosis (Masson trichrome, 100x); **D -** giant cell and feathery changes of the hepatocytes (H&E, 200x).

The gallbladder showed denuded lining epithelium and the presence of an occasional island of hyaline cartilage in its wall (Figure 2A). The hyaline cartilage in the wall of the gallbladder was predominantly mature, apart from an occasional focus showing immature morphology. The wedge of the liver showed a classical picture of distal obstructive cholangiopathy with portal-based fibrosis, occasional porto-portal bridging, exuberant ductular reaction, ductular cholestasis, occasional ductal disarray, and the changes associated with hepatocanalicular cholestasis (Figures 2B-D).

Overall histomorphology was consistent with a distal obstructive cholangiopathy (biliary atresia) with obliterated extrahepatic bile duct and atretic gallbladder. The portal plate at the hilum and the gallbladder contained cartilaginous islands in their walls.

## DISCUSSION

The occurrence of cartilage around the bile duct, porta hepatis, or gallbladder has been documented in a few anecdotal case reports of BA. We document this cartilage in two locations (porta hepatis and gallbladder wall). This represents the second documented case of BA with cartilage around the gallbladder in the English literature and the only one with hyaline cartilage at two locations (Table 1).^1-9^ The corresponding author has the experience of seeing three other cases of BA with cartilage in the extrahepatic biliary tree and the gallbladder wall including two unpublished cases.^1^

**Table 1:** Reported cases of biliary atresia with hyaline cartilage in English literature (partly modified from Ayyanar et al.^1^)

Ref.	Age	Sex	Portal plate/porta hepatis	Bile duct (pericholedochal)	Gallbladder wall	Associated features
Hassab et al.^3^	5 m	F	-	+	-	Multiple congenital anomalies including BASM syndrome
Mirkin and Knisely^4^	14 d	F	+	-	-	-
Mirkin and Knisely^4^	6 w	M	+	-	-	-
Kashiwagi et al.^5^	2 m	F	-	+ (in liver biopsy)	-	CMV+
Stahlschmidt et al.^2^	55 d	F	+	-	-	-
Altamirano and Drut^6^	3 m	NM	-	-	+	-
Vij et al.^7^	2 m	NM	+	-	-	-
Gupta et al.^8^	65 d	NM	+	-	-	-
Ayyanar et al.^1^	3 m	M	-	+	-	CMV+
Elhence and Sinha^9^	5 m	NM	- (?)	-	- (?)	-
IC	2 m	F	**+**	**-**	**+**	-

BASM = Biliary atresia splenic malformation; CMV = Cytomegalovirus; F = female; IC = index case; M = male; NM = Not mentioned; d = days; w = weeks; m = months; + present; - absent; ? not clearly mentioned.

The exact cause and the nature of the cartilage around the proximal extrahepatic biliary tree components in BA is a matter of debate. BA appears to be a multifactorial disease. The etiopathogenesis of BA includes viral (eg: rotavirus, reovirus, cytomegalovirus, Epstein- Barr virus), inflammatory, genetic, ciliary, and immune-mediated causes.^10-12^ The previously published cases of BA with cartilage around the biliary tree do not highlight any common feature to suggest a particular etiology.

Various theories consider the cartilage as a choristoma or an adaptive connective tissue metaplastic response of tissue to injury. Cartilaginous choristomas are characterized by mature hyaline cartilage limited by a thin layer of the perichondrium. The intrahepatic bile ducts, extrahepatic bile ducts, and portal plate are endodermal derivatives. On the contrary, cartilage is mesodermal in origin, indicating it to be a developmental malformation during embryogenesis. Mirkin and Knisely^4^ reported 2 cases of BA with cartilage in the adventitial layer of the common bile duct and proposed the theory of developmental malformation as a possible cause. Stahlschmidt et al.^2^ also considered defective morphogenesis to be the culprit. Adaptive metaplasia, on the other hand, is the transformation of one cell type to another due to chronic injury, inflammation, or insult to the tissue. Metaplastic responses, such as the epithelial-mesenchymal transition triggered by damage to the bile duct epithelium, can result in metaplastic response near epithelial damage.^6^ In that context, histologically, one would expect new immature cartilage formation to merge with the stroma, although they may exhibit more differentiated mature appearing cartilage.^1^ The theory of adaptive metaplasia was further supported by Gupta et al.,^8^ who documented bile ductules exhibiting squamous metaplasia with cartilaginous metaplasia in the gallbladder.

Both metaplastic responses and choristomas may contribute to the scenario. In the index case, there were abrupt well-defined lobules of mature cartilage encased by perichondrium. However, the occasional presence of immature cartilage aligns more with the characteristics of an adaptive connective tissue/ mesenchymal metaplastic response.

## CONCLUSION

Cartilage was present in the portal plate and gallbladder in a child undergoing a portoenterostomy. The immature cartilage, merging the perichondrium with the surrounding soft tissue, suggests a metaplastic etiology. Alternately, this may represent a heterotopia.
